# Severe Organ Impairment Was Common in Elderly Individuals with Dengue in Guangdong, China

**DOI:** 10.4269/ajtmh.24-0023

**Published:** 2024-07-09

**Authors:** Xingyu Leng, Huiqin Yang, Wenxin Hong, Jianfeng He, Jian Wang, Xi He, Lingzhai Zhao, Baolin Liao, Xuefu Chen, Dongying Xie, Jie Peng, Changtai Wang, Jiamin Feng, Lu Liao, Kanghong Jin, Linghua Li, Xiaoping Tang, Chengfeng Qin, Fuchun Zhang

**Affiliations:** ^1^ Guangzhou Medical Research Institute of Infectious Diseases, Department of Infectious Disease, Guangzhou Eighth People’s Hospital, Guangzhou Medical University, Guangzhou, People’s Republic of China;; ^2^ Department of Infectious Diseases, Guangzhou Red Cross Hospital, Guangzhou, People’s Republic of China;; ^3^ Guangdong Provincial Centre for Disease Control and Prevention, Guangzhou, People’s Republic of China;; ^4^ Department of Clinical Laboratory, Guangzhou Eighth People’s Hospital, Guangzhou Medical University, Guangzhou, People’s Republic of China;; ^5^ Department of Infectious Disease, Guangdong Provincial People’s Hospital, Guangdong Academy of Medical Sciences, Guangzhou, People’s Republic of China;; ^6^ Department of Infectious Disease, the Third Affiliated Hospital, Sun Yat Sen University, Guangzhou, People’s Republic of China;; ^7^ Department of Infectious Disease, Nanfang Hospital of Southern Medical University, Guangzhou, People’s Republic of China;; ^8^ Department of Infectious Diseases, Second Affiliated Hospital of Anhui Medical University, Hefei, People’s Republic of China;; ^9^ Institution of Infectious Disease, Guangzhou Eighth People’s Hospital, Guangzhou Medical University, Guangzhou, People’s Republic of China;; ^10^ State Key Laboratory of Pathogen and Biosecurity, Beijing Institute of Microbiology and Epidemiology, Beijing, People’s Republic of China

## Abstract

Guangdong, China, has experienced several dengue epidemics involving thousands of confirmed cases in recent decades, and elderly individuals suffered severe dengue (SD) most seriously. However, the clinical characteristics and risk factors for SD among elderly patients in Guangdong have not been investigated. Patients older than 65 years were recruited and divided into a dengue fever (DF) group and an SD group according to the 2009 Dengue Guidelines of the WHO. We analyzed the clinical manifestations of the elderly patients with dengue and then assessed the risk factors for SD. Of a total of 1,027 patients, 868 patients were diagnosed as having DF and 159 as having SD. Of the 159 elderly patients with SD, 129 (81%) had comorbidities, with hypertension being the most common. Severe organ impairment (SOI) (115, 54%) was the most common presentation in SD patients, followed by severe plasma leakage (52, 24.4%) and severe hemorrhage (46, 21.6%). The most common symptom of SOI was kidney injury, followed by heart injury and central nervous system injury. Furthermore, multivariate regression revealed that the presence of chronic obstructive pulmonary disease (COPD), a lower red blood cell (RBC) count (≤3.5 × 10^12^/L; odds ratio [OR], 0.35; 95% CI, 0.17–0.55; *P <*0.001), lower serum albumin (ALB) (≤35 U/L; OR, 0.18; 95% CI, 0.09–0.32; *P <*0.001), and hyperpyrexia (body temperature ≥39°C; OR, 1.8; 95% CI, 1.2–2.6, *P <*0.001) were risk factors for SD. Severe organ impairment was the predominant manifestation in elderly individuals with SD characterized by kidney injury. The potential risk factors of SD such as presence of COPD and hyperpyrexia and lower RBC and ALB levels might help clinicians identify patients with SD early.

## INTRODUCTION

Dengue fever (DF) is an arboviral disease caused by dengue virus (DENV) and poses a worldwide public health problem.^1^ Currently, dengue is endemic in more than 100 countries in the tropical and subtropical populated areas and is reported predominantly in Southeast Asia, the Americas, and the Western Pacific.^2^ In 2019, the WHO declared dengue as one of the 10 threats to global health.^3^ From 1990 to 2019, the number of incident dengue cases increased by 85.5%, while the number of deaths attributable to dengue increased by 28.1%.^4^ Moreover, most of the dengue disease burden is concentrated in young people aged 15 to 49 years worldwide^4^ but occurs in people aged >14 years or <70 years in regions of endemicity.^5^ In the same period, the number of reported dengue cases increased from 376 in 1990 to over 20,000 in 2019 in mainland China, and the number of reported cases even exceeded 40,000 in 2014.^6^ In addition, patients older than 70 years suffer the highest burden in China.^7^ Between 2005 and 2020, more than 80,000 indigenous DF cases were reported in mainland China, 74% of which were concentrated in Guangdong Province.^8^ Furthermore, in Guangdong, 65% of the severe dengue (SD) patients who died the most often were more than 60 years old.^9^ However, the clinical characteristics and risk factors for SD among elderly patients in China are still unknown.

According to the dengue guidelines published by the WHO in 2009, SD was clinically classified as severe hemorrhage (SH), severe plasma leakage (SPL), or severe organ impairment (SOI).^10^ However, the criteria for organ impairment were indistinct except for liver injury.^9^^,^^11^ Considering that SOI is an important SD classification in mainland China, a precise definition of SOI is urgently needed.

To understand the clinical manifestations and risk factors for SD among elderly individuals, laboratory-confirmed patients with dengue aged above 65 years from 2013 to 2023 were recruited for analysis.

## MATERIALS AND METHODS

### Study design and participants.

This retrospective observational study was based on the Dengue Clinical Research Database of Guangdong Province (DCRDGP). The clinical data used in this study originated mainly from Guangzhou Eighth People’s Hospital, Guangzhou Medical University, Guangzhou Red Cross Hospital, Guangdong Provincial People’s Hospital, Guangdong Academy of Medical Sciences, the Third Affiliated Hospital, Sun Yat-Sen University, and Nanfang Hospital of Southern Medical University. According to the 2009 WHO guidelines, 1,027 laboratory-confirmed elderly patients (aged ≥65 years) with dengue were recruited between 2013 and 2023. Patient demographic information, clinical manifestations, laboratory indicators, and radiographic examination data were collected from the DCRDGP.

The study subjects were divided into a DF group and an SD group according to the 2009 WHO guidelines. All patients with SD were comprehensively evaluated by two or more clinical experts. This study was retrospectively registered in the Chinese Clinical Trial Registry (ChiCTR2100046696). The research protocol was approved by the ethics committee of the Guangzhou Eighth People’s Hospital, Guangzhou Medical University (reference numbers 20100835, 20131224, and 20159264), and written informed consent forms were obtained from all the participants.

### Definition of SOI.

We supplemented the definition of SOI based on the 2009 WHO guidelines. Patients were diagnosed with SOI in accordance with any established criteria. The exclusion criteria for patients were as follows: 1) severe liver involvement: aspartate aminotransferase (AST) and/or alanine aminotransferase (ALT) of ≥1,000 U/L or total bilirubin (TB) of ≥85.5 *µ*mol/L; 2) severe kidney involvement: serum creatinine (sCr) of ≥176.8 *µ*mol/L, sCr that was increased 2-fold and over the upper threshold, or urine output consistently below 0.5 mL/(kg h) for 12 hours; 3) severe heart involvement: acute heart failure (AHF), cardiogenic shock, or severe cardiac arrhythmia; (4) central nervous system (CNS) involvement: encephalopathy, encephalitis, myelitis, or Guillain-Barré syndrome (GBS); (5) others: acute respiratory distress syndrome (ARDS) not due to SPL, rhabdomyolysis, acute pancreatitis, or disseminated intravascular coagulation.^9^^,^^12^

### Diagnostic tests.

RNA was extracted from each serum sample using a QIAamp viral RNA mini kit (Qiagen, Germantown, MD) according to the manufacturer’s instructions. Quantitative reverse transcription-polymerase chain reaction assays were performed to determine specific serotype and viral load (VL) using a dengue detection kit (DaAn, Guangzhou, China). Dengue virus nonstructural protein 1 (NS1) antigen was determined in acute-phase serum samples by enzyme immunoassay (diagnostic kit for dengue virus NS1 antigen; Wantai, Beijing, China).

## STATISTICAL ANALYSES

Continuous variables are summarized as the mean with standard deviation or median with interquartile range (IQR), and categorical variables are summarized as frequencies and proportions. Continuous variables were compared by Student’s *t* test and assessed with the nonparametric Mann-Whitney *U* test in the case of skewed variables. Categorical variables were assessed using the χ2 test or Fisher’s exact test. A multivariate logistic regression model was applied to determine the independent risk factors in accordance with the Wald (forward Wald) method, generating odds ratios (ORs) and 95% CIs. All the statistical analyses were performed with SPSS Statistics 21.0 software (IBM Corp., Armonk, NY). The kinetics of the laboratory parameters were determined using GraphPad Prism 8.3 (GraphPad Software, Inc., La Jolla, CA).

## RESULTS

### Identification of elderly patients with SD.

All patients were diagnosed with dengue if positive for either viral genomic RNA or the NS1 antigen. In total, 1,027 patients were recruited and divided into the SD group (159, 15%) or the DF group (868, 85%), the median age was 74 years (IQR: 68–80), and the SD patients were older than the DF patients (80 years versus 73 years, *P* <0.001). Also, 582 (56.6%) patients were males; there was no significant difference between the sexes (*P =* 0.20) (Table 1).

**Table 1:** Clinical manifestation characteristics of elderly patients with SD

Variables	Total (*N* = 1,027)	DF (868, 85%)	SD (159, 15%)	*P*-Value
Sex, male	582 (56.6%)	477 (56%)	96 (60.4%)	0.20
Age, years, IQR	74 (68, 80)	73 (68, 80)	80 (72.5, 87)	<0.001
Comorbidities	411 (40%)	282 (32%)	129 (81%)	<0.001
Hypertension	311 (31%)	214 (25%)	97 (61%)	<0.001
CVD	116 (12%)	62 (7%)	54 (34%)	<0.001
Diabetes	105 (10%)	66 (8%)	39 (25%)	<0.001
COPD	45 (4%)	21 (3%)	24 (15%)	<0.001
CKD	13 (1%)	5 (0.6%)	8 (5%)	<0.001
Cirrhosis	9 (0.9%)	8 (0.9%)	1 (0.6%)	1.0
Constitutional clinical manifestations				
Duration of onset in days, median, IQR	11 (7–12)	10 (7–13)	13 (9–15)	<0.001
Fever	816 (79%)	685 (67%)	131 (82%)	0.46
Nausea	280 (28%)	243 (29%)	37 (23%)	0.10
Vomiting	205 (20%)	162 (19%)	43 (27%)	0.02
Rash	128 (13%)	106 (12%)	22 (14%)	0.36
Myalgia	386 (38%)	346 (41%)	40 (25%)	<0.001
Headache	340 (28%)	293 (34%)	47 (30%)	0.13
Arthralgia	148 (15%)	129 (15%)	19 (12%)	0.17
Ostealgia	42 (4.2%)	30 (3.5%)	12 (7.5%)	0.02
Diarrhea	162 (16%)	125 (15%)	37 (23%)	<0.001
Anepithymia	457 (45%)	388 (46%)	69 (43%)	0.32
Cough	306 (30%)	223 (26%)	83 (52%)	<0.001
Main laboratory tests, median, IQR				
WBC (10^9^/L)	4.1 (2.2–4.3)	3.8 (2–4.2)	4.7 (2.6–6)	<0.001
Lym (10^9^/L)	1 (0.2–1.2)	0.8 (0.6–1.7)	0.9 (0.5–1.5)	0.83
CRP (mg/L)	12.1 (5.6–36.2)	10.1 (3.6–24)	45.2 (15.7–97)	<0.001
Hb (g/L)	117 (106–127)	118 (107–133)	102 (82–106)	<0.001
RBC (10^12^/L)	4 (3.6–4.3)	3.9 (3.6–4.3)	3.3 (2.8–4)	<0.001
HCT (%)	38.1 (34.6–40.5)	38.4 (35–42.9)	39.2 (33.6–41.9)	0.22
ALB (U/L)	33 (30.3–36)	33.8 (30–36.4)	29 (26–31.5)	<0.001
AST (U/L)	72 (48–109)	71 (46–229.4)	111 (77–355)	<0.001
ALT (U/L)	45 (30–73)	48 (31.2–226.6)	62 (45–125)	0.004
sCr (*µ*mol/L)	75.4 (65–91.2)	80 (68–99.6)	134 (75–182)	<0.001
LDH (U/L)	368 (292–573)	325 (270–458)	821 (415–1,455)	<0.001
BUN (mmol/L)	4.4 (3.4–6.4)	4.6 (3.9–6)	11.5 (5.8–17.9)	<0.001
PLT (10^9^/L)	51 (28–86)	51 (33–136)	20 (13–32)	<0.001
APTT (s)	44.8 (40.1–50.9)	44.8 (40.6–50.8)	45.5 (38.5–52.7)	0.75
INR	0.98 (0.9–17)	1 (0.95–17)	1.08 (0.99–1.52)	<0.001
D-dimer (µg/mL)	1,340 (650–2,385)	1,060 (640–1,920)	2,060 (660–4,400)	0.55
Warning signs				
Abdominal pain	63 (6%)	41 (5%)	22 (14%)	<0.001
Mucosal bleeding	217 (21%)	150 (17%)	69 (43%)	<0.001
Liver enlargement >2 cm	12 (1%)	12 (1%)	0 (0%)	
Restlessness	284 (28%)	219 (26%)	65 (41%)	<0.001
Clinical fluid accumulation*	133 (17%)	65 (9%)	71 (49%)	<0.001

Values are presented as number (%) of cases unless otherwise indicated. ALB = albumin; ALT = alanine aminotransferase; APTT = activated partial thromboplastin time; AST = aspartate aminotransferase; BUN = blood urine nitrogen; CKD = chronic kidney disease; COPD = chronic obstructive pulmonary disease; CRP = C-reactive protein; CVD = cardiovert disease; Hb = hemoglobin; HCT = hematocrit; INR = international normalized ratio; IQR = interquartile range; LDH = lactate dehydrogenase; Lym = lymphocyte; PLT = platelet count; RBC = red blood cell; sCr = serum creatinine; SD = severe dengue; WBC = white blood cell.

* Of all 800 cases that received clinical fluid accumulation examination, 657 cases were dengue fever and 143 cases were SD.

### Clinical manifestations of elderly patients with SD.

The main clinical manifestations of the elderly patients are shown in Table 1. A total of 411 (40%) patients had comorbidities, with hypertension being the most common comorbidity (311, 30%), followed by cardiovascular disease (CVD) (116, 11%) and diabetes (105, 10%). More SD patients than DF patients had preexisting hypertension (61% versus 25%, *P <*0.001), CVD (34% versus 7%, *P <*0.001), diabetes (25% versus 8%, *P <*0.001), chronic obstructive pulmonary disease (COPD) (15% versus 3%, *P <*0.001), and chronic kidney disease (5% versus 0.6%, *P <*0.001). The duration of onset was from the day that the first dengue symptom manifested in a patient to the day the patient became an outpatient or died. The median duration of onset was 11 days. The duration of onset was significantly higher in SD patients than in DF patients (13 versus 10 days, *P <*0.001). Moreover, cough (52% versus 26%, *P <*0.001), vomiting (27% versus 19%, *P =* 0.02), and diarrhea (23% versus 15%, *P <*0.001) were more common in SD patients. Among the 816 patients with fever, double-rise fever (9% versus 5%, *P =* 0.03) and hyperpyrexia (body temperature ≥39°C) (44% versus 30%, *P =* 0.003) were more common in SD patients than DF patients, while fever duration was not significantly different between the SD and DF patients (Supplemental Table 1).

Among the SD patients, significantly lower hemoglobin (Hb) (102 versus 118 g/L, *P <*0.001), red blood cell (RBC) counts (3.3 × 10^12^ versus 3.9 × 10^12^, *P <*0.001), serum albumin (ALB) (29 versus 33.8 U/L, *P <*0.001), and platelets (PLT) (20 × 10^9^ versus 51 × 10^9^/L, *P <*0.001) were detected (*P <*0.001), while higher white blood cell (WBC) counts (4.7 × 10^9^ versus 3.8 × 10^9^/L, *P <*0.001), C-reactive protein (CRP) (45.2 versus 10.1 mg/L, *P <*0.001), AST (111 versus 71 U/L, *P <*0.001), ALT (62 versus 48 U/L, *P <*0.001), serum creatinine (sCr) (134 versus 80 *µ*mol/L, *P <*0.001), lactate dehydrogenase (LDH) (821 versus 325 U/L, *P <*0.001), blood urea nitrogen (BUN) (11.5 versus 4.6 mmol/L, *P <*0.001), and international normalized ratio (INR) (1.08 versus 1, *P <*0.001) were measured. The hematocrit (HCT) was less than 40% in elderly patients, and there was no significant difference between the SD and DF patients (39.2% versus 38.4%, *P =* 0.22) (Table 1).

### Classification of SOI.

According to SD classification of the 2009 WHO guidelines, the most common symptom of SD in elderly patients was SOI (115; 54%), followed by SPL (52; 24.4%) and SH (46; 21.6%) (Table 2). Among the 115 elderly patients with SOI, kidney involvement was the most common manifestation (46, 19.8%), followed by heart involvement (41,17.7%) and CNS involvement (34,14.7%). Forty-three (18.5%) patients had multiple organ dysfunction syndrome (MODS).

**Table 2:** Classification of SOI among elderly patients

Classification	*n* (%)
SD classification	
SOI	115 (54.0)
SPL	52 (24.4)
SH	46 (21.6)
SD subdivision	
SOI only	81 (50.9)
SPL only	21 (13.2)
SH only	15 (9.4)
SOI+SPL	11 (6.9)
SOI+SH	11 (6.9)
SPL+SH	8 (5.1)
SOI+SPL+SH	12 (7.6)
SOI classification	
Acute kidney injury	46 (19.8)
sCr ≥176.8 µmol/L	33 (72)
sCr increased 2-fold and over upper threshold	13 (28)
Acute heart injury	41 (17.7)
Acute heart failure	30 (73)
Serious cardiac arrhythmia	9 (22)
Cardiogenic shock	2 (5)
CNS injury	34 (14.7)
Encephalitis	18 (53)
Encephalopathy	15 (44)
Guillain-Barré syndrome	1 (3)
Liver injury	33 (14.2)
AST ≥1,000 (U/L)	19 (58)
TB ≥ 85.5 (*µ*mol/L)	7 (21)
ALT and AST ≥1,000 (U/L)	5 (15)
ALT ≥1,000 (U/L)	2 (6)
Lung injury	26 (11.2)
ARDS	25 (96)
Serious pneumonia	1 (4)
Others	9 (3.9)
Acute pancreatitis	5 (56)
Rhabdomyolysis	4 (44)
MODS	43 (18.5)

ALT = alanine aminotransferase; ARDS = acute respiratory distress syndrome; AST = aspartate aminotransferase; CNS = central nervous system; MODS = multiple organ dysfunction syndrome; sCr = serum creatinine; SD = severe dengue; SH = severe hemorrhage; SOI = severe organ impairment; SPL = severe plasma leakage; TB = total bilirubin.

The following was found in patients with SOI. 1) Of 46 patients with AKI, 33 patients (72%) had an sCr of ≥176.8 *µ*mol/L, 2 of which had urine output that was consistently below 0.5 mL/(kg h) for 12 hours. Another 13 (28%) patients were diagnosed with a 2-fold increase in sCr and over the upper threshold. 2) Of the 41 patients with heart impairment, 30 (73%) patients were diagnosed with AHF, 9 (22%) patients were diagnosed with severe cardiac arrhythmia, and 2 (5%) patients were diagnosed with cardiogenic shock. 3) Of 34 patients with CNS involvement, 18 (53%) patients presented with encephalitis, 15 (44%) patients presented with encephalopathy, 4 of which experienced acute cerebral infarction, and 1 (3%) patient presented with GBS. 4) Also, 33 patients with liver involvement were diagnosed with acute liver injury, 26 patients (79%) had an AST and/or ALT of ≥1,000 (U/L), and 7 patients (21%) had a TB of ≥85.5 *µ*mol/L, 1 of which had a TB concentration higher than 170 *µ*mol/L. 5) Of 26 patients with lung injury, 25 (96%) patients had ARDS, and 1 (4%) patient had serious pneumonia. 6) Five patients were diagnosed with acute pancreatitis, and 4 patients were diagnosed with rhabdomyolysis.

### Risk factors for SD in elderly patients.

All 1,027 patients with complete data were included in the univariate analysis and multivariate models, and the results showed that the presence of COPD, WBC, RBC, ALB level, and PLT were also associated with SD. We found that COPD (OR, 7.04; 95% CI, 3.81–12.99; *P <*0.001), low RBC count (≤3.5 × 10^12^/L; OR, 0.35; 95% CI, 0.16–0.46.10; *P <*0.001), and low serum ALB levels (≤35 U/L; OR, 0.18; 95% CI, 0.09–0.32; *P <*0.001) were the independent risk factors for SD according to the multivariable models (Table 3). A total of 816 fever patients were selected in the univariate analysis and multivariate models for determining fever indicators in patients with SD. Hyperpyrexia (OR, 1.8; 95% CI, 1.2–2.6; *P <*0.001) was also an independent risk factor (Supplemental Table 2).

**Table 3:** Risk factors for SD among elderly patients

Variables	Univariable Analysis		Multivariable Analysis	
	OR (95% CI)	*P*-Value	OR (95% CI)	*P*-Value
COPD	7.29 (2.40–22.10)	<0.001	7.04 (3.81–12.99)	<0.001
WBC (10^9^/L)	1.35 (0.98–2.01)	0.03		
≥4			1.49 (0.69–3.19)	0.31
<4			1 (ref)	–
RBC (10^12^/L)	0.32 (0.17–0.55)	<0.001		
≤3.5			0.35 (0.16–0.46)	<0.001
>3.5			1 (ref)	–
ALB (U/L)	0.53 (0.32–0.81)	<0.001		
≤35			0.18 (0.09–0.32)	0.001
>35			1 (ref)	–
PLT (10^9^/L)	1.02 (0.96–1.24)	0.04		
≤50			1.65 (1.41–3.99)	0.19
>50			1 (ref)	–

ALB = albumin; COPD = chronic obstructive pulmonary disease; OR = odds ratio; PLT = platelet count; RBC = red blood cell; ref = reference; SD = severe dengue; WBC = white blood cell.

### Kinetics of laboratory parameters in elderly patients with SD.

**Figure 1.**
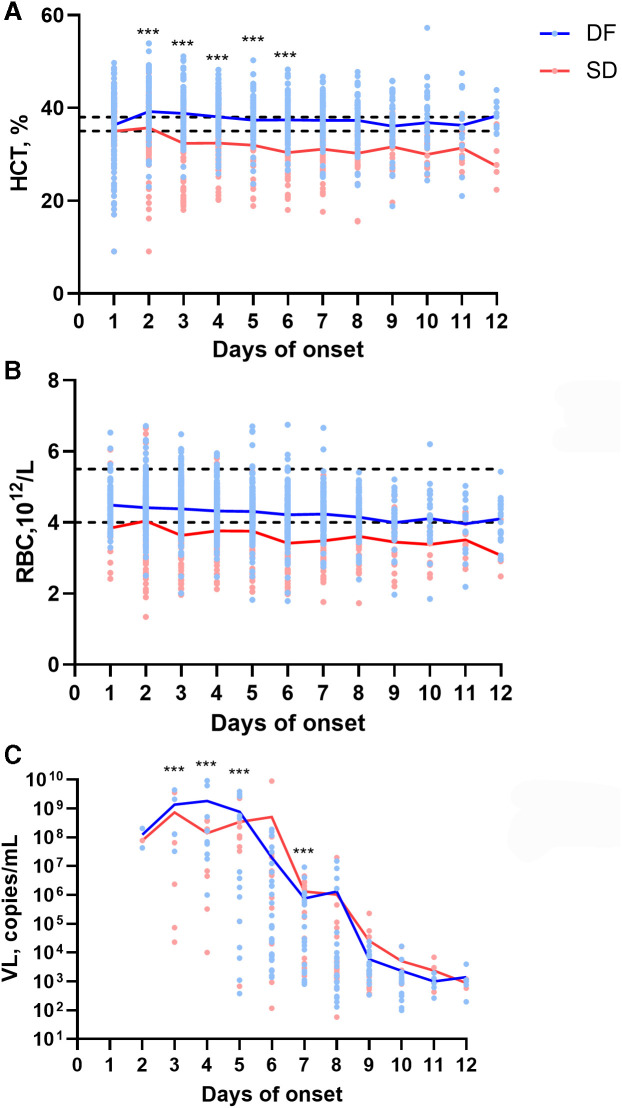
Kinetics of the laboratory parameters during the 12 days of illness. The data are for 1027 elderly patients with dengue. A. HCT, B. RBC and C. serum viral load (VL) were investigated in elderly patients with dengue fever (DF) and severe dengue (SD) during 12 days of illness. The black dotted line represents the normal value. ***, *P* < 0.001.

Red blood cells, HCT, and VL were investigated 12 days after disease onset (Figure 1). Interestingly, the HCT in the SD group was lower than the normal level during the illness, and its trend was the same as that of the RBC count. Furthermore, the HCT was significantly lower between day 2 and day 6 after the onset of the illness in the SD group than in the DF group (*P <*0.001) (Figure 1A). A notable drop in RBC count was observed from the onset of disease, and the RBC levels were maintained at a low level throughout the illness in SD patients. In contrast, the RBC level in DF patients was normal and significantly greater than that in the SD patients (*P <*0.001) (Figure 1B). Moreover, the VL was measured in a total of 264 patients, of which 157 had DF and 107 had SD. The VL of the SD patients was significantly lower than that of the DF patients between the 3rd and 5th days after onset but was greater on the 7th day (*P <*0.001) (Figure 1C).

Furthermore, we confirmed the DENV serotype in 36 patients: 33 (92%) cases were infected with DENV-1, and 3 (8%) cases were infected with DENV-2.

## DISCUSSION

The main manifestations of SD were demonstrated in patients of different age groups. Severe plasma leakage was more likely to be common in pediatric patients with SD.^10^^,^^13^^,^^14^ However, the main presentation of SD in adults is complex. Severe plasma leakage is the most common presentation in Asia,^15^^–^^17^ whereas SH is manifested most frequently in the Americas.^18^ In a few studies on elderly patients with SD, Rowe et al.^19^ reported that SPL was prominently manifested in Singapore, and another study reported that elderly patients tend to have fewer SHs than their younger counterparts.^18^^,^^20^ Lee et al.^21^ found that acute renal failure occurred more frequently in individuals aged 65 years with dengue hemorrhagic fever than in younger adults. Because of the vague definition of SOI in the 2009 WHO guidelines, except for liver involvement, the clinical features of SOI in elderly patients were still unclear. The present study recruited 1,027 elderly patients. Severe organ impairment was the main presentation of SD according to the 2009 WHO guidelines, while the laboratory indicators for organ injury were increased simultaneously. Based on the 2009 WHO guidelines, Kan et al.^16^ distinguished SOI patients using more detailed classifications. However, explicit organ involvement in almost 21.1% of patients was still unknown. More exhaustive criteria of SOI are urgently needed in clinical diagnoses and treatment. In the present study, we further categorized the patients described above according to more precise supplementary definitions of SOI. Acute kidney injury and AHF were likely more common in SOI patients. Among patients with CNS involvement, acute cerebral infarction occurred without cerebral comorbidities. Severe organ impairment in elderly patients was characterized by multiple organ injuries at the same time, which may potentially be associated with the disease spectrum of hypertension and CVD as the main comorbidities. In this study, we further supplemented the diagnostic criteria for AKI by laboratory tests and added moderate jaundice to the criteria for liver injury. Acute heart failure, serious cardiac arrhythmia, and cardiogenic shock were included in the heart impairment criteria; encephalitis, encephalopathy, and Guillain-Barré syndrome were included in the CNS impairment criteria; and ARDS not due to plasma leakage and serious pneumonia were included in the lung impairment criteria. We also considered acute pancreatitis and rhabdomyolysis as SOIs. In addition, MODS was also diagnosed as an SOI. More comprehensive diagnostic criteria of SOI would be helpful for clinical application.

Preexisting chronic comorbidities like diabetes, hypertension, obesity, and cardiovascular diseases were considered risk factors for SD.^22^ The high morbidity of diabetes makes it common in patients with dengue, and a number of these patients are at risk for SD in low and middle-income countries.^23^ In addition, COPD was a risk factor for SD among the elderly patients in the present study. Furthermore, a higher frequency of COPD was found in elderly patients with dengue hemorrhagic fever who died.^21^ The effect of COPD on SD pathogenesis in elderly patients should be explored. In Guangzhou, the most frequent comorbidity was hypertension, followed by CVD and diabetes. Though hypertension was not found to be a risk factor for SD among elderly patients, we still consider it associated with SD and even SOI. Further studies are necessary to seek the connection between SD and hypertension in vivo and in vitro.

The mechanism of SD is still unknown, and previous studies have hypothesized that cell apoptosis, host metabolism, endothelial activation, virus transcriptomics, and inflammatory and antibody-dependent enhancement may be potential mechanisms.^24^ To understand the features of HCT, RBC count, and VL between SD and DF, dynamic changes of those indicators during illness were investigated. Considering that the median duration of onset in elderly patients was 11 days, detection of indicators occurred in few patients after 12 days of onset. To obtain dinamic change of those indicators, the laboratory test data including the first 12 days of onset were selected. From day 3 to day 5 of onset, the VL of SD patients was significantly lower than that of DF patients. But on day 7 of onset, the VL of SD patients was significantly lower than that of DF patients. There were no differences in VL between the two groups except for on days 3, 4, 5, and 7. It is well known that high VL is a risk factor for SD in pediatric patients.^25^^,^^26^ However, a higher VL was more frequently measured in elderly patients with DF. In this study, we did not consider that VL was directly associated with SD among elderly patients. Considering that DENV-1 was the predominant serotype in Guangdong Province,^27^^,^^28^ we confirmed the DENV serotypes of some SD patients. Most patients were infected with DENV-1, but the relationship between DENV-1 and SD could not be assessed effectively due to lack of sufficient data. More attention should be given to the role of the SD mechanism in elderly people.

Interestingly, in our study, we found that HCT levels were decreased in elderly patients with SD, which was significantly different from what has been observed in patients with typical SD.^10^ The reasons for the decreased HCT levels in elderly patients with SD should be explored.

Multivariate analysis identified low RBC and ALB levels as independent risk factors for SD. However, because the RBC level was significantly lower in SD patients than in DF patients throughout the entire illness, RBC level was not a good predictor. Our previous study suggested that the baseline serum ALB concentration is a prognostic indicator of SD,^29^ which was also shown in a meta-analysis by Sangkaew et al.^30^

In view of poor immunological senescence, reduced physiological functions, and effects of comorbidities, the clinical management of elderly patients with SD should differ from that of young individuals. Unfortunately, the current management strategies are extrapolated from the general population owing to the scarcity of studies on elderly patients. In this study, we found that the serum ALB concentration was an independent risk factor for SD. Along with atypical HCT, we suggest that the serum ALB concentration may be a valuable indicator of disease severity in elderly patients with SD. The clinical management of elderly patients with dengue is necessary.

Our study has several limitations. First, because of the retrospective study design, not all laboratory tests, including indicators of coagulation and laboratory tests of bacterial infection, were performed for all patients. Therefore, their role might be underestimated in patients with increased WBC counts during SD. Second, the lack of complementary radiographic data made clinical fluid accumulation investigations difficult. Third, serum VL and DENV serotypes were confirmed in only a few patients because of a lack of serum samples, and the effect of DENV on SD in elderly individuals is still unclear. Lastly, because of poor serum samples for detection of DENV IgG and IgM, the effect of immune status on SD is unknown.

To the best of our knowledge, this is the largest retrospective multicenter study among elderly patients with SD. Based on it, we found that SOI was the predominant manifestation, characterized by AKI and AHF, in elderly patients with SD. Moreover, the potential risk factors for SD, which are COPD, ALB level lower than 35 U/L, RBC level lower than 3.5 × 10^12^/L, and hyperpyrexia, could help clinicians identify patients with SD at an early stage. Our study, which provides critical implications for the clinical management of SD, might be useful for admission of elderly patients with SD.

## Supplemental Materials

10.4269/ajtmh.24-0023 Supplemental Materials
