# Supplementary material for: Severe Organ Impairment Was Common in Elderly Individuals with Dengue in Guangdong, China
Source: Am J Trop Med Hyg. 2024 Jul 9;111(3):610–6. doi: 10.4269/ajtmh.24-0023 (PMC11376186; doi:10.4269/ajtmh.24-0023)
Supplement: Supplemental Materials [file tpmd240023.SD1.pdf]

supplementary Table 1 Features of fever among elderly patients with severe dengue

Variables	Total (n=816)	DF (685, 83.95%)	SD (131,16.05%)	*p* value
Fever duration	6 (4,7)	5 (4,7)	6 (4,8)	0.08
Double-rise fever	53 (6.5%)	39 (5%)	14 (9%)	0.03
< 39℃	553 (68%)	479 (70%)	74 (56%)	0.003
≥ 39℃	263 (32%)	206 (30%)	57 (44%)	

supplementary Table 2 Risk factors of fever type for severe dengue among elderly patients

Variables	Univariable analysis		Multivariable analysis	
	OR(95% CI）	*p* value	OR(95% CI）	*p* value
Double-rise fever	1.4 (0.7,2.8)	0.4		
Hyperpyrexia	1.7 (1.1,2.5)	0.02	1.8 (1.2,2.6)	<0.001

NOTE: Hyperpyrexia was body temperature ≥ 39°C
